# Nationwide Trends and Outcomes of Alcohol Use Disorders in COPD Hospitalizations in Spain, 2016–2023

**DOI:** 10.3390/jcm14176045

**Published:** 2025-08-26

**Authors:** Teresa Gómez-Garcia, Rodrigo Jiménez-Garcia, Valentín Hernández-Barrera, Ana López-de-Andrés, David Carabantes-Alarcon, Ana Jiménez-Sierra, Elena Labajo-González, Andrés Bodas-Pinedo, Javier de-Miguel-Diez

**Affiliations:** 1 Respiratory Care Department, Hospital General Universitario Gregorio Marañón, Instituto de Investigación Sanitaria Gregorio Marañón (IiSGM), Universidad Complutense de Madrid, 28007 Madrid, Spain; t.gomez@salud.madrid.org (T.G.-G.); javier.miguel@salud.madrid.org (J.d.-M.-D.); 2 Department of Public Health and Maternal & Child Health, Faculty of Medicine, Universidad Complutense de Madrid, IdISSC, 28040 Madrid, Spain; rodrijim@ucm.es (R.J.-G.); abodas@ucm.es (A.B.-P.); 3 Preventive Medicine and Public Health Teaching and Research Unit, Health Sciences Faculty, Universidad Rey Juan Carlos, 28922 Madrid, Spain; valentin.hernandez@urjc.es; 4 Department of Public Health and Maternal & Child Health, Faculty of Pharmacy, Universidad Complutense de Madrid, IdISSC, 28040 Madrid, Spain; anailo04@ucm.es; 5 Faculty of Medicine, Universidad San Pablo Ceu, 28668 Madrid, Spain; a.jimenez100@usp.ceu.es; 6 Department of Legal Medicine, Psychiatry and Pathology, Faculty of Medicine, Universidad Complutense de Madrid, 28040 Madrid, Spain; melabajo@ucm.es

**Keywords:** chronic obstructive pulmonary disease, COPD, alcohol use disorder, psychiatric comorbidities prevalence, substance use hospitalization, mortality, sex

## Abstract

**Background:** Chronic obstructive pulmonary disease (COPD) is a prevalent condition with high morbidity and mortality, often accompanied by comorbidities such as alcohol use disorder (AUD). A thorough understanding of the interaction between COPD and AUD is crucial for improving patient outcomes and addressing management challenges. **Objectives:** This study analyzed temporal trends, clinical characteristics, and hospital outcomes associated with AUD among adults hospitalized with COPD in Spain between 2016 and 2023. **Methods:** A population-based cohort study was conducted using the Spanish Hospital Discharge Registry. We included adults aged ≥40 years with a diagnosis of COPD. AUD was identified through ICD-10 codes. Temporal trends in AUD prevalence were evaluated using Joinpoint regression, stratified by sex. We also assessed clinical characteristics including pneumonia, obesity, asthma, obstructive sleep apnea (OSA), supplemental oxygen use, long-term steroid use, and mechanical ventilation. Outcomes analyzed included ICU admission and in-hospital mortality (IHM). **Results:** Among 2,545,151 COPD hospitalizations, 263,568 (10.35%) had an AUD diagnosis. AUD prevalence rose from 8.66% in 2016 to 12.57% in 2023, with a sharper increase in women. Patients with AUD were younger and had higher rates of tobacco use (84.11% vs. 49.33%; *p* < 0.001) and psychiatric disorders. Multivariable analysis showed male sex, substance use, psychiatric illness, and external cause admissions were independently associated with AUD. Although overall IHM was lower in AUD patients (7.46% vs. 8.2%; *p* < 0.001), it increased with age, pneumonia, COVID-19, and higher comorbidity. IHM rose progressively, peaking in 2023 (15.6%). **Conclusions:** AUD prevalence in COPD hospitalizations increased significantly, especially in women. IHM also rose over time. These results highlight the need for integrated approaches targeting mental health and substance use in COPD management.

## 1. Introduction

Chronic Obstructive Pulmonary Disease (COPD) is a progressive respiratory condition characterized by persistent airflow limitation and an enhanced chronic inflammatory response in the airways and lungs, primarily due to exposure to noxious particles or gases such as tobacco smoke [1]. It is a widespread and debilitating illness, affecting approximately 11.8% of the Spanish population aged 40 and older, according to the EPISCAN II [2] study, conducted in 2017, and is associated with significant morbidity and mortality. Notably, over 80% of COPD patients are estimated to have at least one comorbid chronic condition. The most prevalent comorbidities include cardiovascular diseases, diabetes mellitus, osteoporosis, musculoskeletal disorders, and mental health conditions such as anxiety and depression [3]. Multimorbidity in COPD not only increases hospital admissions in terms of frequency and duration [4] but also drives extensive direct medical costs such as emergency visits, medications, and long-term oxygen therapy, as well as significant indirect costs, including reduced work productivity, premature retirement, impaired quality of life, and substantial caregiver burden [5].

Within this context, patients hospitalized with COPD frequently present with concurrent mental and behavioral disorders due to substance abuse (MBDA) [6,7]. MBDA describes a spectrum of psychiatric conditions directly caused by the harmful or dependent use of psychoactive substances. This includes both substance use disorders (persistent and problematic patterns of misuse leading to clinical impairment) and substance-induced disorders, which manifest as psychiatric symptoms during periods of intoxication or withdrawal [8]. Alcohol Use Disorder (AUD) is a prevalent condition in the general population, affecting approximately 13.9% of U.S. adults annually, according to DSM-5 criteria in the NESARC-III study [9]. The prevalence of AUD among COPD patients varies across studies, influenced by multiple factors including study design, population demographics, and diagnostic criteria [10,11]. While some studies report lower prevalence rates, others indicate higher rates, particularly among specific subgroups such as older males with psychological distress. AUD in COPD patients is characterized by distinct demographic profiles and socioeconomic vulnerabilities [6]. Its presence leads to more severe inpatient complications, elevated costs, and greater likelihood of readmission, often worsening outcomes when combined with other comorbidities [6].

AUD may influence the development and progression of Chronic Obstructive Pulmonary Disease (COPD) through both biological and behavioral mechanisms. Biologically, chronic alcohol consumption impairs lung immunity by suppressing alveolar macrophage function, increasing oxidative stress, and disrupting the integrity of the pulmonary epithelial barrier [12]. These effects collectively heighten susceptibility to respiratory infections and may accelerate COPD progression. Indirectly, AUD is strongly associated with other substance use disorders, most notably tobacco dependence, which remains the leading etiological factor for COPD. In addition, elevated rates of illicit drug use (e.g., cocaine, cannabinoids, opioids) among individuals with AUD further compromise respiratory health and may contribute to airway inflammation or acute exacerbations.

Chronic and heavy alcohol consumption adversely affects COPD by compromising multiple pulmonary defense mechanisms. Long-term ethanol exposure impairs mucociliary clearance by disrupting ciliary beating and CFTR-mediated airway hydration, thereby increasing susceptibility to pathogen colonization and infection [13]. Concurrently, alcohol induces significant oxidative stress and depletes alveolar glutathione—key antioxidant protection in the lung—leading to diminished alveolar macrophage function and weakened epithelial barrier integrity, which further impairs pulmonary host defense and potentiates airway injury and COPD progression [14]. Overall, chronic heavy drinking appears to exacerbate COPD symptoms by increasing the frequency of respiratory infections, accelerating decline in lung function, and elevating the risk of exacerbations and mortality.

Understanding the multifaceted nature of COPD and its associated conditions is crucial for improving patient outcomes and optimizing healthcare resource allocation. The intersection of COPD with AUD introduces unique challenges in patient management, affecting clinical presentation, treatment adherence, and overall prognosis. This study aims to analyze retrospectively temporal trends, clinical characteristics, and hospital outcomes associated with AUD among adults hospitalized with COPD in Spain between 2016 and 2023.

## 2. Materials and Methods

We conducted a population-based observational descriptive study using data from the Spanish National Hospital Discharge Database (SNHDD), managed by the Spanish Ministry of Health. The SNHDD is a mandatory administrative registry that compiles information on all hospital discharges from public and private hospitals in Spain. It includes only inpatient admissions and excludes data from patients treated exclusively in emergency departments.

The database contains detailed information on patient demographics (age, sex), admission and discharge dates, discharge destination (recovery, transfer, or in-hospital death), primary and up to 19 secondary diagnoses, up to 20 diagnostic or therapeutic procedures, and admission to intensive care units (ICU). Diagnoses and procedures are coded using the International Classification of Diseases, 10th Revision, Clinical Modification (ICD-10-CM) [15].

The study period ranged from 1 January 2016, to 31 December 2023, and included all hospital discharges from public hospitals in Spain. The study population consisted of individuals aged ≥40 years with a diagnosis of COPD, identified by the presence of ICD-10 code J40 to J44 and J47 in any of the 20 diagnostic fields. We excluded those with missing data on sex, admission or discharge dates, or discharge status.

The inclusion criteria for this study were as follows: adults aged ≥40 years; patients with a diagnosis of chronic obstructive pulmonary disease (COPD), identified by the presence of ICD-10 codes J40–J44 and J47 in any of the 20 diagnostic fields; and all hospital discharges from public hospitals in Spain between 1 January 2016, and 31 December 2023. Only inpatient admissions were considered.

The exclusion criteria were: missing data on sex, admission or discharge dates, or discharge status; and patients treated exclusively in emergency departments, as SNHDD only collects information on inpatient hospitalizations.

The primary outcome was the presence of AUD, defined by the ICD-10 code F10 in any diagnostic position. The analysis was stratified by sex. Covariates included year of admission, age (as a continuous variable and in categorized age groups), and hospitalization characteristics such as ICU admission and in-hospital mortality (IHM).

To assess comorbidity, we applied the Charlson Comorbidity Index, which includes the following conditions: acute myocardial infarction, congestive heart failure, peripheral vascular disease, cerebral vascular disease, dementia, pulmonary disease, connective tissue disease, peptic ulcer disease, liver disease, diabetes, diabetes with complications, hemiplegia or paraplegia, renal disease, cancer, metastatic cancer, severe liver disease, HIV disease [16]. Specific clinical conditions of interest included obesity, depression, anxiety disorders, specific personality disorders, and external causes of morbidity and mortality. We also analyzed the presence of pneumonia, obesity, asthma, obstructive sleeping apnea (OSA), the use of supplemental oxygen, long term use of steroids and mechanical ventilation during hospitalization. Additionally, other substance use disorders were evaluated, including tobacco, cocaine use, cannabis use, and opioid dependence. Since 2020, the presence of COVID-19 was also considered. Regarding the outcomes of hospital admission of the subjects included in the study, we analyzed the admission to ICU and IHM.

All ICD-10 codes used to define exposure and covariates are detailed in Table S1.

### 2.1. Statistical Analysis

The study population was described by year and stratified by sex. Quantitative variables were summarized using means and standard deviations when normally distributed (normality assessed using the Kolmogorov–Smirnov test) and as medians and interquartile ranges (IQR) when non-normally distributed. Categorical variables were presented as frequencies and percentages.

Temporal trends in the prevalence of AUD among hospitalizations COPD were assessed separately for men and women using Joinpoint regression analysis. This method allows for the identification of statistically significant changes in trend patterns and estimates the annual percent change (APC) over time [17].

For other covariates, temporal trends were evaluated using linear regression for continuous variables, the Jonckheere–Terpstra test for non-normally distributed variables, and the Cochran–Mantel–Haenszel test for categorical variables.

In the bivariable analysis, comparisons between groups were made using Student’s *t*-test for normally distributed continuous variables, the Mann–Whitney U test for non-normally distributed variables, and Fisher’s exact test for categorical variables. Where applicable, *p*-values were adjusted for multiple comparisons using the Bonferroni–Holm correction method.

To identify independent factors associated with AUD among patients with COPD, we performed unconditional multivariable logistic regression using a generalized logit function, stratified by sex. The same multivariable approach was used to evaluate factors independently associated with in-hospital mortality among patients with both COPD and AUD. Only variables showing a statistically significant association in the bivariable analysis were considered in the multivariable models. Model development followed the strategy described by Hosmer and colleagues [18]. Results were expressed as adjusted odds ratios (aORs) with 95% confidence intervals (CIs).

All statistical analyses were conducted using Stata software, version 14 (StataCorp, College Station, TX, USA). A two-tailed *p*-value < 0.05 was considered statistically significant.

### 2.2. Ethical Considerations

This study was conducted using data from the SNHDD, an administrative dataset owned and maintained by the Spanish Ministry of Health. Access to the SNHDD requires submission of a formal request specifying the research objectives and methodology. The Ministry evaluates each request from both a scientific and ethical standpoint before authorizing the release of the data.

The dataset provided for this study was completely anonymized and did not contain any personally identifiable information. As the data were obtained for research purposes in an anonymous and aggregate format, and in accordance with Spanish legislation governing the secondary use of health data, ethical approval from a clinical research ethics committee was not required.

Moreover, the database was made available to the research team free of charge, and its use complied fully with national regulations on the protection of personal data and the ethical standards of epidemiological research.

The Spanish legislation is available at: Ley 14/2007; de 3 de julio, de investigación biomédica [19]. Also, according to this law, as this is an administrative database, informed consent is not necessary.

## 3. Results

**Figure 1:**
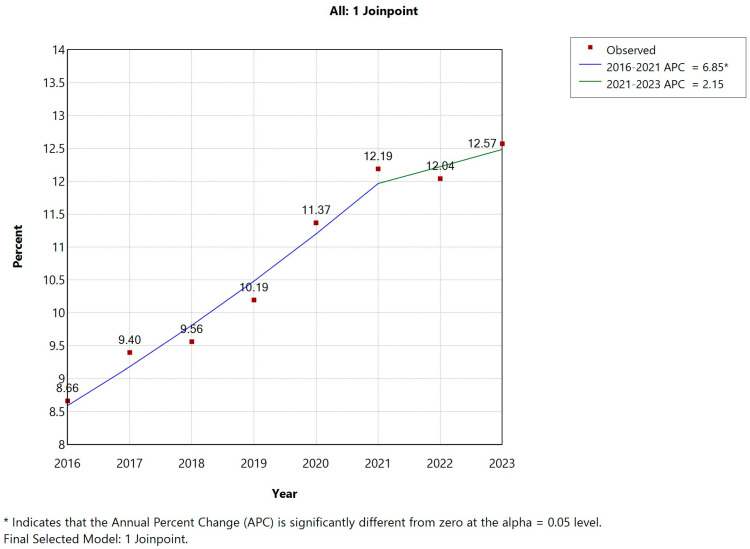
Joinpoint regression for the prevalence of mental and behavioral disorders due to use of alcohol (AUD) over all COPD hospital admissions in Spain (2016–2023).

**Figure 2:**
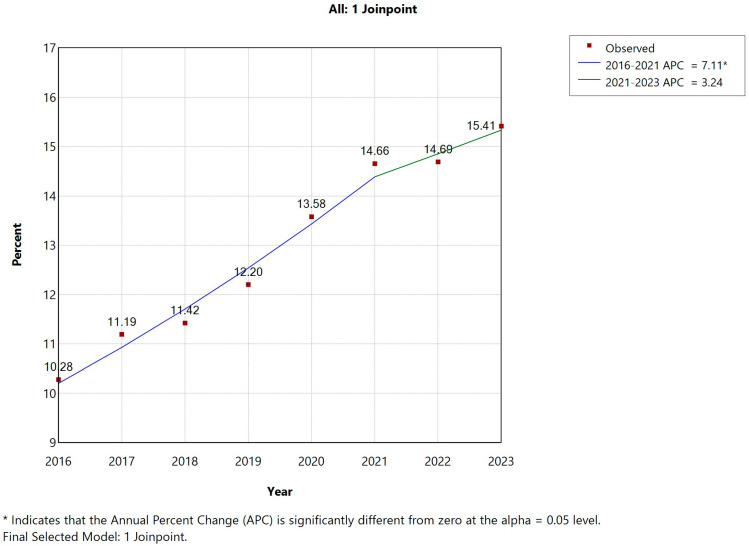
Joinpoint regression for the prevalence of mental and behavioral disorders due to use of alcohol (AUD) in men with COPD hospital admissions in Spain (2016–2023).

**Figure 3:**
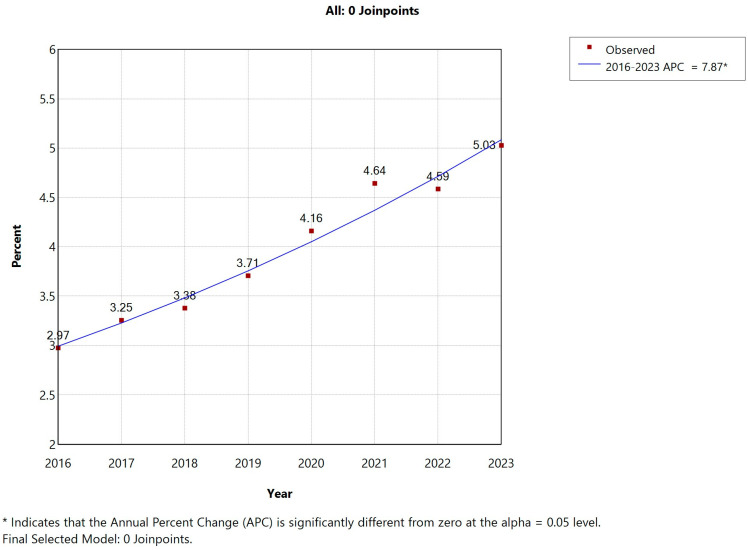
Joinpoint regression for the prevalence of mental and behavioral disorders due to use of alcohol (AUD) in women with COPD hospital admissions in Spain (2016–2023).

Between 2016 and 2023, a total of 2,545,151 hospitalizations involving patients with COPD were recorded in Spain, of which 263,568 (10.35%) included a co-diagnosis of AUD. Joinpoint regression analysis revealed a statistically significant increase in AUD prevalence among hospitalized COPD patients between 2016 and 2021, rising from 8.66% to 12.26%. Although the upward trend appeared to continue through 2023, the increase was no longer statistically significant after 2021 (Figure 1, Figure 2 and Figure 3).

Stratified by sex, this trend was more pronounced in men, whose AUD prevalence rose from 10.28% in 2016 to 15.29% in 2021 (APC: 7.11%, *p* < 0.05), with no significant change thereafter. Among women, despite a lower baseline prevalence, AUD still increased significantly from 2.97% to 5.03% over the full study period (APC: 7.87%, *p* < 0.05) (Figure 3). In 2016, men accounted for 92.41% of AUD cases among COPD hospitalizations; this proportion gradually declined to 89.05% by 2023, reflecting an increasing representation of women—from 7.59% in 2016 to 10.95% in 2023 (Table 1). This shift indicates a significant rise in female AUD cases (*p* < 0.001). Meanwhile, the mean age of COPD patients with AUD increased significantly during the study period, rising from 67.68 to 69.18 years (*p* < 0.001), with particularly notable growth observed in the 50–65 and 65–80 year age brackets.

The clinical characteristics and hospital outcomes of COPD patients with AUD—summarized in Table 1—reveal a notable rise in substance use from 2016 to 2023. Cocaine use experienced the most pronounced increase, doubling from 2.49% to 4.86%. In the same period, cannabis use climbed from 2.03% to 3.47%, opioid dependence grew from 2.40% to 3.27%, and tobacco consumption increased modestly from 82.5% to 84.73% (*p* < 0.001 for all). Most comorbidities also trended upward—pneumonia, sleep apnea, obesity, anxiety, personality disorders, and admissions for external causes all showed statistically significant increases (*p* < 0.001)—whereas the prevalence of depression remained unchanged (*p* = 0.465). The Charlson Comorbidity Index rose modestly from 1.10 to 1.16 (*p* < 0.001). COVID 19 prevalence increased from 2.83% in 2020 to 9.48% in 2022, ICU admissions rose slightly (7.12% → 7.79%), and IHM peaked in 2020 (8.48%) then decreased to 7.34%, with an overall significant upward trend (*p* < 0.001) (Table 1).

Comparing COPD patients with versus without AUD (Table 2), those with AUD were significantly younger (68.44 vs. 75.44 years; *p* < 0.001) and had higher prevalences of substance use—cocaine (3.69% vs. 0.45%), cannabinoids (2.79% vs. 0.34%), opioids (2.95% vs. 0.65%), and tobacco (84.11% vs. 49.33%) (all *p* < 0.001)—along with more frequent psychiatric comorbidities (depression 4.44% vs. 4.15%; anxiety 4.02% vs. 3.57%; personality disorders 1.62% vs. 0.42%). Those with AUD had lower rates of pneumonia (10.42% vs. 11.23%) and asthma (2.67% vs. 4.73%), but higher prevalences of sleep apnea (13.19% vs. 12.66%) and obesity (14.52% vs. 12.78%). However, among women with AUD, sleep apnea (7.93% vs. 9.43%) and obesity (14.05% vs. 16.73%) were lower than in their non AUD counterparts (*p* < 0.001). The overall Charlson comorbidity Index was slightly higher in AUD patients (1.15 vs. 1.13; *p* < 0.001), though women with AUD had a lower index than women without (0.98 vs. 1.03; *p* < 0.001). COVID 19 was less frequent among AUD patients (3.01% vs. 3.54%). ICU admission rates were higher (7.25% vs. 5.8%), especially in women (7.82% vs. 5.23%), while IHM was lower in the AUD group (7.46% vs. 8.20%) across sexes.

Regarding sex differences among AUD patients (Table S2), men (*n* = 248,836) were older than women (*n* = 24,732; 68.93 vs. 63.45 years; *p* < 0.001). Women showed higher prevalences of cocaine (5.92% vs. 3.47%), cannabinoids (4.39% vs. 2.63%), and opioids (5.26% vs. 2.73%) (*p* < 0.001), whereas tobacco use was slightly higher among men (84.12% vs. 83.96%; *p* < 0.001). Psychiatric conditions—depression (12.18% vs. 3.67%), anxiety (11.47% vs. 3.27%), and personality disorders (6.2% vs. 1.16%)—occurred more frequently in women (*p* < 0.001), and external-cause admissions were also higher (7.74% vs. 5.24%; *p* < 0.001). Men had higher Charlson Comorbidity Index scores (1.15 vs. 0.98; *p* < 0.001), as well as greater prevalence of asthma, obesity, sleep apnea, pneumonia, and COVID 19 diagnoses, while ICU admission was more frequent in women (7.82% vs. 7.2%; *p* < 0.001) and IHM was higher in men (7.63% vs. 5.76%). Female patients received more intensive interventions—corticosteroids, supplemental oxygen, invasive and non invasive ventilation (all *p* < 0.001).

Multivariable logistic regression (Table 3) identified several independent predictors of AUD. Using the 40–50 year age group as the reference, the aOR for the 50–65 year group was 1.09 in men and 0.95 in women. In the 65–80 year group, odds decreased to 0.60 for men and 0.54 for women, while those aged ≥80 showed further reductions (men: aOR 0.25; women: aOR 0.15). These findings indicate that the likelihood of AUD increases modestly in middle-aged men but declines progressively in older age groups across both sexes. Tobacco conferred the highest AUD risk (aOR 3.95 in men, 4.84 in women), followed by cocaine, cannabinoids, and opioids. Psychiatric disorders (personality disorders aOR 2.04/3.35; depression aOR 1.34; anxiety aOR 1.21), external causes (aOR 1.55), higher Charlson comorbidity Index (1.20/1.27), and obesity in men (aOR 1.11) were significant predictors. AUD odds increased annually, reaching aORs of 1.40 (men) and 1.32 (women) in 2023, with male sex strongly associated (aOR 3.54).

The cohort was stratified by IHM status (Table 4), revealing that patients who died during admission were significantly older (mean age 71.74 vs. 68.17 years; *p* < 0.001), with mortality rates rising sharply with age and reaching 23.82% in those aged ≥80 years. Non-survivors exhibited lower rates of substance use and comorbid conditions such as asthma, sleep apnea, and obesity (*p* < 0.001). Conversely, they had higher prevalences of pneumonia, ICU admission (18.29% vs. 6.36%), COVID-19 infection (5.22% vs. 2.84%), and increased need for supplemental oxygen, invasive ventilation (11.59% vs. 1.71%), and non-invasive ventilation (8.44% vs. 4.01%). IHM escalated from 8.81% in 2016 to 15.6% in 2023, with the highest rate observed in women (17.96%).

Finally, predictors of IHM (Table 5) showed that age was the strongest risk factor (with women ≥ 80 years showing aOR 4.72), along with pneumonia (aOR 1.41), COVID 19 (aOR 1.80), and higher CCI (aOR 1.30). Protective associations were observed for obesity (aOR 0.66), sleep apnea (aOR 0.67), depression/anxiety (aOR 0.81), and personality disorders (aOR 0.76). Mortality risk was elevated during the COVID years of 2020 (aOR 1.23) and 2021 (aOR 1.14), without further increase afterward, and male sex modestly increased mortality risk (aOR 1.06).

## 4. Discussion

In our study, we found that between 2016 and 2023, 10.35% of patients hospitalized with COPD in Spain had a co-diagnosis of AUD. Moreover, the prevalence of AUD among COPD inpatients increased steadily over this period. These findings are consistent with data from U.S. inpatient cohorts. For instance, a nationwide review of COPD and asthma admissions (2012–2015) reported a documented AUD prevalence of 4.1%, with AUD associated with greater respiratory failure, increased need for mechanical ventilation, prolonged hospital stay, and higher 30-day readmission rates [6]. Similarly, a U.S. emergency department study found that 2.11% of visits among COPD/asthma patients involved alcohol misuse; these individuals were older (mean age 58.1 vs. 53.7), predominantly male (69.5%), and faced elevated rates of respiratory failure and ventilation [6]. More pertinent to our population, the 2022 EDADES survey revealed that 76.5% of adults aged 15–64 in Spain had consumed alcohol in the past year. Notably, 10.5% reported daily alcohol consumption, and 14.7% had experienced binge drinking in the past year [20]. This underscores a high baseline prevalence of alcohol use in Spain. Regarding the impact of the COVID-19 pandemic, Oliván-Blázquez et al. reported a substantial worsening of alcohol use disorder during the first year of the pandemic in Spain, based on primary care electronic records [21]. Although the study did not specifically evaluate respiratory diagnoses, the observed exacerbation in alcohol misuse during this period highlights a potential indirect relationship with chronic respiratory diseases such as chronic bronchitis and COPD. Despite these valuable epidemiological insights, to our knowledge, there is a lack of European or Spanish studies specifically assessing AUD among COPD patients in hospital or primary-care settings. This underscores the urgent need for further research to address this significant interaction between alcohol misuse and chronic respiratory diseases.

In our cohort, male patients continued to predominate; however, the rate of increase in prevalence over time was notably steeper among women, resulting in a narrowing of the gender gap. Although men represented the majority of cases, the proportion of women affected grew increasingly over the study period. Patients with AUD were, on average, several years younger than their non-AUD counterparts, and the likelihood of AUD decreased with advancing age, particularly in older age brackets relative to middle-aged adults. Regarding sex- and age-related dynamics, our data not only mirror U.S. patterns in male predominance and younger age of AUD patients but also reveal a pronounced demographic shift: the accelerated rise of AUD in female COPD patients, despite their low initial prevalence, highlights a trend that pre-existing literature has not fully addressed.

The accelerated rise of AUD among women and its higher prevalence in younger populations can be attributed to both sociocultural shifts and biological vulnerabilities. Over the past two decades, changing gender roles and greater social acceptability of female drinking have contributed to narrowing sex gaps in alcohol consumption. These sociocultural changes not only influence drinking behaviors but may also interact with inherent biological susceptibilities, further intensifying the risk in women. Women develop AUD more rapidly than men, a pattern known as “telescoping”, and experience greater physical harm at lower levels of alcohol intake. This is largely attributed to biological differences, including lower gastric alcohol dehydrogenase activity, less total body water, and higher body fat percentage, which lead to higher blood alcohol concentrations. As a result, women are more vulnerable to alcohol-related liver disease, cardiovascular complications, and neurocognitive damage. Psychosocial factors such as greater exposure to internalizing disorders and stress-related drinking further compound this risk [22].

The observed decline in AUD probability with advancing age is consistent with general population trends showing lower substance use among older adults. For instance, a study utilizing data from the National Comorbidity Survey Replication found that the prevalence of 12-month substance use disorders was lower in older adults (65 years and older) compared to younger age groups, with 0% prevalence for any substance use disorder in the 65 and older group [23]. The lower prevalence of AUD in older adults may be attributed to various factors, including age-related physiological changes that reduce alcohol tolerance, social role changes such as retirement and social isolation, and potential underdiagnosis due to overlapping symptoms with other age-related conditions [24].

The high prevalence of tobacco consumption (84.02%) among patients with AUD in our cohort underscores the significant overlap between substance use disorders. Moreover, our analysis also indicates that substance use was markedly higher in the AUD group compared to those without AUD across all categories. Specifically, cocaine use was 3.69% in the AUD group versus 0.45% in the non-AUD group; cannabinoid use was 4.39% versus 1.88%; and opioid dependence was 5.26% versus 1.83%. These findings align with previous research indicating that individuals with AUD are more likely to engage in other substance use behaviors. For example, a study found that individuals in opioid antagonist treatment had a higher prevalence of substance misuse, including tobacco, alcohol, and illicit drugs, compared to those without substance misuse [25].

The fact that patients diagnosed with AUD frequently exhibit higher prevalence of use of other substances, including cocaine, cannabis, opioids, and notably tobacco, can be largely understood through the lens of self-medication and syndrome overlap. The self-medication hypothesis posits that these individuals may turn to alcohol and other psychoactive substances in attempts to alleviate distressing mood or anxiety symptoms; over time, such coping behaviors can evolve into independent substance use disorders [26].

In addition, the concept of polysubstance use highlights the inherent comorbidity and overlapping risk profiles among substance use disorders. Tobacco use, for instance, often co-occurs with alcohol dependence, with cross-sensitization and shared reinforcement pathways increasing vulnerability to multiple substances [27]. Further, socioeconomic disadvantages associated with AUD, such as reduced access to care, financial stress, and poor treatment adherence, can exacerbate psychiatric symptoms and reduce resilience, indirectly encouraging broader substance use as coping. The convergence of these factors, self-medication, psychiatric comorbidity, and socioeconomic vulnerability, offers a coherent explanation for why individuals with AUD demonstrate elevated use of other substances.

Regarding sex differences within the AUD group, our data show that cocaine use was more prevalent among women (5.92% vs. 3.47%), as was cannabinoid use (4.39% vs. 2.63%) and opioid dependence (5.26% vs. 2.73%). These findings are consistent with studies indicating that women with AUD may be more likely to use certain substances compared to their male counterparts. For instance, a study by Grischott et al. [28] found that women in Opioid Agonist Treatment (OAT) had a higher prevalence of substance misuse, including tobacco, alcohol, and illicit drugs, compared to men. A key explanation for these sex-specific differences lies in the higher burden of psychiatric comorbidities observed in women with AUD. Internalizing disorders like depression and anxiety are strongly associated with AUD, and individuals may resort to alcohol as a form of self-medication. This concept can be extended to explain the increased use of other substances by women with AUD, as they might be employing these additional substances to manage a broader spectrum of underlying psychological distress or psychiatric symptoms.

The data from our study indicate a significant increase in the prevalence of various comorbidities among patients with AUD hospitalized for COPD between 2016 and 2023. Notably, psychiatric disorders such as depression, anxiety, and personality disorders were more prevalent in the AUD group compared to non-AUD patients. These findings align with existing literature. So, a systematic review and meta-analysis reported that COPD patients have a significantly higher prevalence of psychiatric comorbidities, including depression and anxiety, compared to non-COPD individuals. The odds ratios for these conditions ranged from 1.78 to 1.96, indicating a substantial association [29]. Moreover, a study conducted in Spain found that COPD patients exhibited higher rates of mental disorders, psychological distress, and psychiatric medication use compared to controls [30]. The significant association between AUD and various substance use and psychiatric comorbidities is as well supported by prior studies. Internalizing disorders such as depression and anxiety are strongly associated with AUD. Individuals with these conditions may use alcohol as a form of self-medication, leading to a higher risk of developing AUD [31]. The slight increase in the Charlson Comorbidity Index over time in our study is consistent with findings from other research indicating that the burden of comorbidities in COPD patients tends to rise with disease progression [32].

Conversely, conditions like pneumonia and asthma were less common in the AUD cohort. This contrasts with some studies that have reported higher rates of respiratory infections and comorbid asthma among individuals with substance use disorders [33,34]. However, the specific relationship between AUD and these conditions in the context of COPD requires further investigation. Additionally, the lower incidence of COVID-19 among AUD patients in our study may reflect differences in healthcare access, comorbidity profiles, or other factors that warrant further exploration.

Between 2016 and 2023 in Spain, ICU admissions among COPD patients increased slightly, with those diagnosed with AUD exhibiting greater ICU resource use—particularly women. Despite this heightened ICU utilization, in-hospital mortality paradoxically remained lower among AUD patients across both sexes. This suggests that although AUD is linked with more intensive care needs, it does not necessarily drive higher mortality, possibly due to differences in age profiles, illness severity, or care pathways within this subgroup.

International literature examining ICU outcomes in patients with AUD mirrors our conclusions regarding intensive care utilization but often indicates higher mortality. A prospective cohort study in Scotland found that 34.4% of ICU admissions involved patients with AUD, and alcohol dependence was independently associated with significantly greater odds of ICU mortality (OR 2.28) and hospital mortality (OR 2.43) after adjustment for lifestyle factors and age [35]. In U.S. populations, COPD and asthma patients with AUD had higher rates of respiratory failure (OR 1.32) and need for mechanical ventilation in emergency settings, as well as increased ICU admissions, lengthier hospital stays, and greater likelihood of 30 day readmission [6]. Together, these studies indicate that AUD heightens the risk of severe critical illness and intensive care resource use; however, our finding of lower in-hospital mortality among AUD-afflicted COPD patients—especially women—suggests that patient demographics, clinical profiles, and treatment pathways may modulate outcomes.

Multivariate modeling identified several significant determinants of in hospital mortality among COPD patients with alcohol use disorder. Advanced age, especially in elderly women, constituted the most substantial mortality risk. Coexisting infections, most notably pneumonia and COVID 19, as well as a greater burden of comorbidities, significantly elevated the likelihood of in hospital death. Literature largely supports our multivariate findings: advanced age, pneumonia, COVID 19 infection, and comorbidity burden notably increase mortality risk [36,37,38]. Conversely, some chronic conditions, including obesity, sleep apnea, and various psychiatric disorders, were associated with reduced mortality risk.

The observed protective effect of obesity aligns with the well-documented “obesity paradox” in COPD, whereby overweight and obese individuals hospitalized for exacerbations exhibit lower adjusted in-hospital and long-term mortality rates [39]. This paradox has been supported by several large-scale studies, which suggest that excess body mass may confer metabolic and nutritional reserves that help buffer against the catabolic stress of acute illness, particularly in elderly patients [40]. Furthermore, obese patients may receive earlier or more aggressive medical attention due to heightened clinical vigilance, potentially improving outcomes.

Similarly, the association between obstructive sleep apnea (OSA) and reduced in-hospital mortality in COPD patients may, in part, be attributed to its frequent co-occurrence with obesity and increased clinical recognition. Patients with diagnosed OSA are more likely to receive targeted interventions such as CPAP therapy, closer monitoring, and multidisciplinary care, which can mitigate acute deterioration during hospital stays.

Finally, the association of psychiatric comorbidities (e.g., depression, anxiety, personality disorders) with lower in-hospital mortality likely reflects higher levels of healthcare engagement. Patients with diagnosed psychiatric disorders often have more frequent contact with clinical services, may adhere better to follow-up and maintenance therapies, and benefit from systematic monitoring, which could translate into improved acute outcomes [41,42]. While direct mechanistic studies are limited, such patterns of enhanced access and continuity of care offer plausible explanations for survival advantages in this subgroup.

We observed that the probability of AUD decreases with increasing age, while male sex serves as a strong independent predictor. The observed decline in AUD prevalence with advancing age aligns with national surveillance data across populations [43]. Male predominance in AUD risk, often showing roughly 3–4 times higher odds, is well documented in multivariable analyses [44].

Taken together, and building on our findings, alcohol use should be regarded as a potential treatable trait in hospitalized COPD patients, reflecting its rising prevalence and impact on ICU utilization, substance comorbidity, and outcomes. This proposal aligns with GesEPOC 2021’s fourth pillar of COPD care, which advocates for the systematic identification and management of treatable traits (clinical, physiological, or biological characteristics with targeted interventions) alongside exacerbation and risk stratification pathways [45]. Embedding alcohol use assessment and, when indicated, early brief interventions or referrals within COPD treatment protocols enables a more individualized, holistic care approach that integrates respiratory and addiction management, thereby optimizing both pulmonary and overall patient outcomes.

One of the key strengths of this study lies in its extensive sample size, encompassing a large and nationally representative cohort of COPD patients. This broad representation enhances the robustness of the analysis and supports the validity and generalizability of the findings across the national population. However, our study, utilizing the SNHDD, also presents several limitations inherent to administrative health data. Firstly, the reliance on ICD-10 coding for diagnosis classification may introduce inaccuracies due to coding errors, misclassification, or underreporting, especially for conditions not directly related to the primary reason for hospitalization. However, this methodology has previously been used to identify patients with both AUD [46,47] and COPD [48,49]. Additionally, the absence of clinical details such as disease severity, laboratory results, or medication adherence limits the depth of our analyses, potentially leading to residual confounding. Moreover, the observational nature of the study precludes causal inferences, and the lack of post-discharge data restricts our understanding of long-term outcomes and the dynamics between alcohol use disorder and chronic obstructive pulmonary disease. Lastly, variations in coding practices and data collection standards across different hospitals may introduce inconsistencies, impacting the overall validity and comparability of the data.

## 5. Conclusions

Our study reveals a concerning upward trend in the prevalence of AUD among hospitalized COPD patients in Spain, particularly among women. This demographic shift underscores the need for gender-sensitive approaches in both clinical care and public health strategies. The high comorbidity between AUD and other substance use disorders, such as tobacco, cocaine, cannabis, and opioids, highlights the complex interplay between these conditions and the necessity for integrated treatment plans. Additionally, the increased prevalence of psychiatric comorbidities, including depression, anxiety, and personality disorders, among AUD patients suggests a multifaceted pathophysiology that warrants comprehensive management. Despite greater utilization of intensive care resources, the paradoxical lower in-hospital mortality rates among AUD patients may reflect differences in patient demographics and disease severity, pointing to the importance of individualized care approaches.

Therefore, we recommend implementing routine, systematic screening for AUD within COPD treatment protocols to facilitate early identification and tailored interventions, aligning with the “treatable traits” framework. Furthermore, given the disproportionate increase in AUD and psychiatric comorbidities observed specifically among women with COPD, healthcare strategies must evolve to incorporate gender-sensitive approaches, including specialized assessment tools and integrated therapeutic interventions, to improve engagement in care and optimize outcomes for this vulnerable subgroup.

## Figures and Tables

**Table 1:** Number, gender, age, and clinical characteristics and hospital outcomes of alcohol use disorders (AUD) in adults with COPD in Spain (2016–2023).

		2016	2017	2018	2019	2020	2021	2022	2023	*p* for Trend
		N (%)	N (%)	N (%)	N (%)	N (%)	N (%)	N (%)	N (%)	
Number of COPD hospital admissions		303,406	323,500	334,768	333,415	282,377	291,073	331,539	345,073	NA
Number of AUD		26,288 (8.66)	30,399 (9.4)	32,009 (9.56)	33,989 (10.19)	32,103 (11.37)	35,481 (12.19)	39,918 (12.04)	43,381 (12.57)	<0.001
Gender	Male	24,292 (92.41)	28,018 (92.17)	29,394 (91.83)	31,068 (91.41)	29,347 (91.42)	32,154 (90.62)	35,932 (90.01)	38,631 (89.05)	<0.001
	Female	1996 (7.59)	2381 (7.83)	2615 (8.17)	2921 (8.59)	2756 (8.58)	3327 (9.38)	3986 (9.99)	4750 (10.95)	
Age in years. Mean (SD)		67.68 (10.7)	68.07 (10.64)	67.88 (10.51)	68.11 (10.52)	68.33 (10.46)	68.73 (10.34)	68.94 (10.36)	69.18 (10.22)	<0.001
Age groups (years)	40–49	1224 (18.78)	1344 (20.38)	1317 (20.02)	1294 (20.42)	1139 (21.07)	1109 (21.1)	1141 (20.56)	1102 (19.15)	<0.001
	50–64	8891 (17.73)	9880 (18.57)	10,830 (18.68)	11,299 (19.54)	10,530 (21.13)	11,375 (22.27)	12,618 (22.07)	13,393 (22.19)	
	65–79	12,099 (9.35)	14,294 (10.47)	15,064 (10.64)	16,277 (11.33)	15,525 (12.69)	17,417 (13.79)	19,753 (13.49)	21,902 (14.11)	
	≥80	4074 (3.47)	4881 (3.84)	4798 (3.73)	5119 (4.07)	4909 (4.68)	5580 (5.15)	6406 (5.24)	6984 (5.64)	
Cocaine use		655 (2.49)	790 (2.6)	949 (2.96)	1158 (3.41)	1235 (3.85)	1401 (3.95)	1806 (4.52)	2109 (4.86)	<0.001
Cannabinoids use		533 (2.03)	638 (2.1)	687 (2.15)	889 (2.62)	1008 (3.14)	1035 (2.92)	1328 (3.33)	1507 (3.47)	<0.001
Opioid dependence		632 (2.4)	719 (2.37)	885 (2.76)	1023 (3.01)	978 (3.05)	1154 (3.25)	1273 (3.19)	1419 (3.27)	<0.001
Tobacco use		21,688 (82.5)	25,501 (83.89)	26,687 (83.37)	28,744 (84.57)	27,108 (84.44)	29,808 (84.01)	33,801 (84.68)	36,758 (84.73)	<0.001
CCI, Mean (SD)		1.35 (1.1)	1.38 (1.12)	1.41 (1.13)	1.45 (1.14)	1.5 (1.16)	1.54 (1.17)	1.48 (1.16)	1.48 (1.16)	<0.001
Pneumonia		2893 (11.01)	3305 (10.87)	3516 (10.98)	3716 (10.93)	3252 (10.13)	2954 (8.33)	3882 (9.72)	4981 (11.48)	<0.001
Asthma		388 (1.48)	590 (1.94)	824 (2.57)	835 (2.46)	925 (2.88)	1089 (3.07)	1235 (3.09)	1415 (3.26)	<0.001
Sleep apnea		3014 (11.47)	3612 (11.88)	4030 (12.59)	4524 (13.31)	4218 (13.14)	4989 (14.06)	5617 (14.07)	6069 (13.99)	<0.001
Obesity		3484 (13.25)	4075 (13.41)	4341 (13.56)	4773 (14.04)	4710 (14.67)	5685 (16.02)	6058 (15.18)	6583 (15.17)	<0.001
Depression		1170 (4.45)	1331 (4.38)	1367 (4.27)	1485 (4.37)	1470 (4.58)	1543 (4.35)	1790 (4.48)	1978 (4.56)	0.467
Anxiety		611 (2.32)	813 (2.67)	1130 (3.53)	1260 (3.71)	1358 (4.23)	1633 (4.6)	1915 (4.8)	2266 (5.22)	<0.001
Personality disorders		357 (1.36)	420 (1.38)	450 (1.41)	460 (1.35)	594 (1.85)	592 (1.67)	745 (1.87)	814 (1.88)	<0.001
External causes		1249 (4.75)	1442 (4.74)	1641 (5.13)	1786 (5.25)	1824 (5.68)	2085 (5.88)	2277 (5.7)	2653 (6.12)	<0.001
COVID-19		0 (0)	0 (0)	0 (0)	0 (0)	910 (2.83)	1723 (4.86)	3784 (9.48)	1828 (4.21)	<0.001
Long-term use of steroid		437 (1.66)	613 (2.02)	901 (2.81)	1281 (3.77)	1102 (3.43)	1462 (4.12)	1772 (4.44)	2148 (4.95)	<0.001
Supplemental oxygen		2698 (10.26)	3365 (11.07)	3726 (11.64)	3903 (11.48)	3428 (10.68)	3759 (10.59)	4412 (11.05)	4742 (10.93)	<0.001
Invasive ventilation		685 (2.61)	779 (2.56)	815 (2.55)	804 (2.37)	790 (2.46)	842 (2.37)	943 (2.36)	1031 (2.38)	0.207
Non-Invasive ventilation		765 (2.91)	929 (3.06)	1357 (4.24)	1481 (4.36)	1369 (4.26)	1712 (4.83)	2001 (5.01)	2261 (5.21)	<0.001
Admission to ICU		1872 (7.12)	2102 (6.91)	2381 (7.44)	2543 (7.48)	2177 (6.78)	2426 (6.84)	2956 (7.41)	3380 (7.79)	<0.001
IHM		1798 (6.84)	2161 (7.11)	2284 (7.14)	2397 (7.05)	2722 (8.48)	2870 (8.09)	2998 (7.51)	3184 (7.34)	<0.001

COPD: chronic obstructive pulmonary disease. AUD: alcohol use disorders. CCI: Charlson comorbidity index. External causes included ICD 10 codes for accidents, Injury and Intentional self-harm (See Table S1). ICU: intensive care unit. IHM: in-hospital mortality. NA: not available. *p* value for time trend.

**Table 2:** Clinical characteristics and hospital outcomes in adults with COPD, with and without alcohol use disorders (AUD) in Spain according to gender (2016–2023).

	BOTH GENDER			MALE			FEMALE		
	Not AUD	AUD	*p*	Not AUD	AUD	*p*	Not AUD	AUD	*p*
Age Mean (SD)	75.44 (10.97)	68.44 (10.46)	<0.001	75.84 (10.44)	68.93 (10.39)	<0.001	74.3 (12.28)	63.45 (9.91)	<0.001
40–49 years, *n* (%)	38,321 (1.69)	9670 (3.53)	<0.001	22,857 (1.36)	7867 (3.16)	<0.001	15,464 (2.62)	1803 (7.29)	<0.001
50–64 years, *n* (%)	348,788 (15.35)	88,816 (32.47)		225,403 (13.41)	76,749 (30.84)		123,385 (20.88)	12,067 (48.79)	
65–79, *n* (%) years	969,269 (42.67)	132,331 (48.37)		746,983 (44.45)	122,988 (49.43)		222,286 (37.61)	9343 (37.78)	
≥80, *n* (%)	915,205 (40.29)	42,751 (15.63)		685,277 (40.78)	41,232 (16.57)		229,928 (38.9)	1519 (6.14)	
Cocaine use, *n* (%)	10,130 (0.45)	10,103 (3.69)	<0.001	7618 (0.45)	8639 (3.47)	<0.001	2512 (0.42)	1464 (5.92)	<0.001
Cannabinoids use, *n* (%)	7650 (0.34)	7625 (2.79)	<0.001	6120 (0.36)	6539 (2.63)	<0.001	1530 (0.26)	1086 (4.39)	<0.001
Opioid dependence, *n* (%)	14,743 (0.65)	8083 (2.95)	<0.001	10,767 (0.64)	6781 (2.73)	<0.001	3976 (0.67)	1302 (5.26)	<0.001
Tobacco use, *n* (%)	1,120,608 (49.33)	230,095 (84.11)	<0.001	897,417 (53.4)	209,329 (84.12)	<0.001	223,191 (37.76)	20,766 (83.96)	<0.001
CCI, Mean (SD)	1.29 (1.13)	1.46 (1.15)	<0.001	1.38 (1.15)	1.49 (1.15)	<0.001	1.05 (1.03)	1.07 (0.98)	<0.001
Pneumonia, *n* (%)	255,109 (11.23)	28,499 (10.42)	<0.001	196,531 (11.69)	26,187 (10.52)	<0.001	58,578 (9.91)	2312 (9.35)	0.004
Asthma, *n* (%)	107,344 (4.73)	7301 (2.67)	<0.001	43,047 (2.56)	5526 (2.22)	<0.001	64,297 (10.88)	1775 (7.18)	<0.001
Sleep apnea, *n* (%)	287,675 (12.66)	36,073 (13.19)	<0.001	231,971 (13.8)	34,111 (13.71)	0.198	55,704 (9.42)	1962 (7.93)	<0.001
Obesity, *n* (%)	290,283 (12.78)	39,709 (14.52)	<0.001	191,380 (11.39)	36,235 (14.56)	<0.001	98,903 (16.73)	3474 (14.05)	<0.001
Depression, *n* (%)	94,203 (4.15)	12,134 (4.44)	<0.001	45,007 (2.68)	9121 (3.67)	<0.001	49,196 (8.32)	3013 (12.18)	<0.001
Anxiety, *n* (%)	81,174 (3.57)	10,986 (4.02)	<0.001	36,251 (2.16)	8149 (3.27)	<0.001	44,923 (7.6)	2837 (11.47)	<0.001
Personality disorders, *n* (%)	9527 (0.42)	4432 (1.62)	<0.001	4749 (0.28)	2898 (1.16)	<0.001	4778 (0.81)	1534 (6.2)	<0.001
External causes, *n* (%)	114,980 (5.06)	14,957 (5.47)	<0.001	75,326 (4.48)	13,042 (5.24)	<0.001	39,654 (6.71)	1915 (7.74)	<0.001
COVID 19, *n* (%)	80,402 (3.54)	8245 (3.01)	<0.001	59,780 (3.56)	7594 (3.05)	<0.001	20,622 (3.49)	651 (2.63)	<0.001
Long-term use of steroid, *n* (%)	77,218 (3.4)	9716 (3.55)	<0.001	53,408 (3.18)	8681 (3.49)	<0.001	23,810 (4.03)	1035 (4.18)	0.220
Supplemental oxygen, *n* (%)	287,707 (12.67)	30,033 (10.98)	<0.001	209,354 (12.46)	26,982 (10.84)	<0.001	78,353 (13.26)	3051 (12.34)	<0.001
Invasive mechanical ventilation, *n* (%)	37,436 (1.65)	6689 (2.45)	<0.001	27,827 (1.66)	5927 (2.38)	<0.001	9609 (1.63)	762 (3.08)	<0.001
Non-invasive mechanical ventilation, *n* (%)	86,260 (3.8)	11,875 (4.34)	<0.001	59,662 (3.55)	10,372 (4.17)	<0.001	26,598 (4.5)	1503 (6.08)	<0.001
Admission to ICU *n* (%)	131,686 (5.8)	19,837 (7.25)	<0.001	100,750 (6)	17,904 (7.2)	<0.001	30,936 (5.23)	1933 (7.82)	<0.001
IHM, *n* (%)	186,241 (8.2)	20,414 (7.46)	<0.001	144,731 (8.61)	18,989 (7.63)	<0.001	41,510 (7.02)	1425 (5.76)	<0.001

COPD: chronic obstructive pulmonary disease. AUD: alcohol use disorders. CCI: Charlson comorbidity index. External causes included ICD 10 codes for accidents, Injury and Intentional self-harm (See Table S1). ICU: intensive care unit. IHM: in-hospital mortality. *p* value for time trend.

**Table 3:** Multivariable analysis of study variables associated with alcohol use disorders (AUD) in adults with COPD in Spain, according to gender (2016–2023).

		BOTH GENDER	MALE	FEMALE
Study Variable	Categories	OR (95%CI)	OR (95%CI)	OR (95%CI)
Age groups (years)	40–49	Reference	Reference	Reference
	50–64	1.06 (1.03–1.08)	1.09 (1.06–1.13)	0.95 (0.89–1)
	65–79	0.58 (0.57–0.6)	0.6 (0.58–0.62)	0.54 (0.51–0.58)
	≥80	0.24 (0.23–0.24)	0.25 (0.24–0.26)	0.15 (0.14–0.16)
Cocaine use		2.6 (2.51–2.69)	2.54 (2.45–2.64)	2.69 (2.47–2.94)
Cannabinoids use		2.32 (2.23–2.41)	2.17 (2.08–2.26)	3.44 (3.13–3.77)
Opioid dependence		1.12 (1.09–1.16)	1.07 (1.03–1.11)	1.41 (1.3–1.53)
Tobacco use		4.06 (4.02–4.11)	3.95 (3.9–3.99)	4.84 (4.66–5.02)
CCI		1.21 (1.2–1.21)	1.2 (1.2–1.21)	1.27 (1.26–1.29)
Asthma		0.8 (0.78–0.82)	0.82 (0.8–0.85)	0.76 (0.72–0.8)
Sleep apnea		0.84 (0.83–0.85)	0.84 (0.83–0.85)	0.78 (0.75–0.82)
Obesity		1.06 (1.05–1.07)	1.11 (1.1–1.13)	0.72 (0.69–0.75)
Depression		1.34 (1.31–1.36)	1.31 (1.28–1.34)	1.42 (1.36–1.48)
Anxiety		1.21 (1.18–1.24)	1.22 (1.19–1.25)	1.19 (1.14–1.24)
Personality disorders		2.49 (2.39–2.59)	2.04 (1.94–2.15)	3.35 (3.14–3.58)
External causes		1.55 (1.53–1.58)	1.52 (1.49–1.55)	1.85 (1.76–1.95)
COVID 19		0.86 (0.83–0.88)	0.86 (0.84–0.89)	0.78 (0.72–0.85)
Long-term use of steroid		1.02 (1–1.04)	1.03 (1.01–1.06)	0.94 (0.88–1)
Supplemental oxygen		0.88 (0.86–0.89)	0.87 (0.86–0.89)	0.91 (0.87–0.95)
Year of admission	2016	Reference	Reference	Reference
	2017	1.06 (1.04–1.08)	1.06 (1.04–1.09)	1.03 (0.97–1.1)
	2018	1.07 (1.05–1.09)	1.07 (1.05–1.09)	1.01 (0.95–1.07)
	2019	1.1 (1.08–1.12)	1.11 (1.08–1.13)	1.06 (1–1.13)
	2020	1.24 (1.21–1.26)	1.24 (1.22–1.27)	1.17 (1.1–1.24)
	2021	1.35 (1.33–1.38)	1.36 (1.33–1.38)	1.29 (1.22–1.37)
	2022	1.35 (1.33–1.38)	1.36 (1.34–1.39)	1.25 (1.18–1.32)
	2023	1.4 (1.37–1.42)	1.4 (1.38–1.43)	1.32 (1.25–1.4)
Gender	Female	Reference	Reference	Reference
	Male	3.54 (3.49–3.6)	NA	NA

OR: Odds Ratio. CI: Confidence Interval. CCI: Charlson comorbidity index. NA: Not available.

**Table 4:** Clinical characteristics and hospital outcomes in alcohol use disorders (AUD) in adults with COPD, in Spain, according to in hospital mortality and gender (2016–2023).

	BOTH GENDER			MALE			FEMALE		
	NOT IHM	IHM	*p*	NOT IHM	IHM	*p*	NOT IHM	IHM	*p*
Age Mean (SD)	68.17 (10.44)	71.74 (10.14)	<0.001	68.67 (10.37)	72.1 (10.06)	<0.001	63.23 (9.86)	67.04 (9.96)	<0.001
40–49 years, *n* (%)	9413 (3.72)	257 (1.26)	<0.001	7648 (3.33)	219 (1.15)	<0.001	1765 (7.57)	38 (2.67)	<0.001
50–64 years, *n* (%)	84,027 (33.19)	4789 (23.46)		72,515 (31.55)	4234 (22.3)		11,512 (49.39)	555 (38.95)	
65–79, *n* (%) years	121,825 (48.12)	10,506 (51.46)		113,151 (49.23)	9837 (51.8)		8674 (37.22)	669 (46.95)	
≥80, *n* (%)	37,889 (14.97)	4862 (23.82)		36,533 (15.89)	4699 (24.75)		1356 (5.82)	163 (11.44)	
Cocaine use, *n* (%)	9761 (3.86)	342 (1.68)	<0.001	8324 (3.62)	315 (1.66)	<0.001	1437 (6.17)	27 (1.89)	<0.001
Cannabinoids use, *n* (%)	7336 (2.9)	289 (1.42)	<0.001	6271 (2.73)	268 (1.41)	<0.001	1065 (4.57)	21 (1.47)	<0.001
Opioid dependence, *n* (%)	7769 (3.07)	314 (1.54)	<0.001	6494 (2.83)	287 (1.51)	<0.001	1275 (5.47)	27 (1.89)	<0.001
Tobacco use, *n* (%)	213,821 (84.46)	16,274 (79.72)	<0.001	194,173 (84.48)	15,156 (79.81)	<0.001	19,648 (84.3)	1118 (78.46)	<0.001
CCI, Mean (SD)	1.43 (1.14)	1.81 (1.15)	<0.001	1.46 (1.15)	1.84 (1.16)	<0.001	1.05 (0.98)	1.43 (0.98)	<0.001
Pneumonia, *n* (%)	25,386 (10.03)	3113 (15.25)	<0.001	23,289 (10.13)	2898 (15.26)	<0.001	2097 (9)	215 (15.09)	<0.001
Asthma, *n* (%)	6932 (2.74)	369 (1.81)	<0.001	5233 (2.28)	293 (1.54)	<0.001	1699 (7.29)	76 (5.33)	0.005
Sleep apnea, *n* (%)	34,128 (13.48)	1945 (9.53)	<0.001	32,255 (14.03)	1856 (9.77)	<0.001	1873 (8.04)	89 (6.25)	0.015
Obesity, *n* (%)	37,566 (14.84)	2143 (10.5)	<0.001	34,260 (14.91)	1975 (10.4)	<0.001	3306 (14.18)	168 (11.79)	0.012
Depression, *n* (%)	11,470 (4.53)	664 (3.25)	<0.001	8589 (3.74)	532 (2.8)	<0.001	2881 (12.36)	132 (9.26)	<0.001
Anxiety, *n* (%)	10,424 (4.12)	562 (2.75)	<0.001	7702 (3.35)	447 (2.35)	<0.001	2722 (11.68)	115 (8.07)	<0.001
Personality disorders, *n* (%)	4273 (1.69)	159 (0.78)	<0.001	2769 (1.2)	129 (0.68)	<0.001	1504 (6.45)	30 (2.11)	<0.001
External causes, *n* (%)	13,902 (5.49)	1055 (5.17)	0.050	12,075 (5.25)	967 (5.09)	0.338	1827 (7.84)	88 (6.18)	0.023
COVID 19, *n* (%)	7180 (2.84)	1065 (5.22)	<0.001	6595 (2.87)	999 (5.26)	<0.001	585 (2.51)	66 (4.63)	<0.001
Long-term use of steroid, *n* (%)	9076 (3.59)	640 (3.14)	<0.001	8091 (3.52)	590 (3.11)	0.003	985 (4.23)	50 (3.51)	0.189
Supplemental oxygen, *n* (%)	27,342 (10.8)	2691 (13.18)	<0.001	24,498 (10.66)	2484 (13.08)	<0.001	2844 (12.2)	207 (14.53)	0.01
Invasive mechanical ventilation, *n* (%)	4322 (1.71)	2367 (11.59)	<0.001	3813 (1.66)	2114 (11.13)	<0.001	509 (2.18)	253 (17.75)	<0.001
Non-invasive mechanical ventilation, *n* (%)	10,152 (4.01)	1723 (8.44)	<0.001	8810 (3.83)	1562 (8.23)	<0.001	1342 (5.76)	161 (11.3)	<0.001
Admission to ICU *n* (%)	16,104 (6.36)	3733 (18.29)	<0.001	14,542 (6.33)	3362 (17.7)	<0.001	1562 (6.7)	371 (26.04)	<0.001
2016, *n* (%)	24,490 (9.67)	1798 (8.81)	<0.001	22,597 (9.83)	1695 (8.93)	<0.001	1893 (8.12)	103 (7.23)	0.118
2017, *n* (%)	28,238 (11.15)	2161 (10.59)		26,007 (11.31)	2011 (10.59)		2231 (9.57)	150 (10.53)	
2018, *n* (%)	29,725 (11.74)	2284 (11.19)		27,253 (11.86)	2141 (11.27)		2472 (10.61)	143 (10.04)	
2019, *n* (%)	31,592 (12.48)	2397 (11.74)		28,819 (12.54)	2249 (11.84)		2773 (11.9)	148 (10.39)	
2020, *n* (%)	29,381 (11.61)	2722 (13.33)		26,800 (11.66)	2547 (13.41)		2581 (11.07)	175 (12.28)	
2021, *n* (%)	32,611 (12.88)	2870 (14.06)		29,498 (12.83)	2656 (13.99)		3113 (13.36)	214 (15.02)	
2022, *n* (%)	36,920 (14.58)	2998 (14.69)		33,170 (14.43)	2762 (14.55)		3750 (16.09)	236 (16.56)	
2023, *n* (%)	40,197 (15.88)	3184 (15.6)		35,703 (15.53)	2928 (15.42)		4494 (19.28)	256 (17.96)	

COPD: chronic obstructive pulmonary disease. AUD: alcohol use disorders. CCI: Charlson comorbidity index. External causes included ICD 10 codes for accidents, Injury and Intentional self-harm (See Table S1). ICU: intensive care unit. IHM: in-hospital mortality. *p* value for time trend.

**Table 5:** Multivariable analysis of study variables associated with in hospital mortality in alcohol use disorders (AUD) in adults with COPD in Spain, according to gender (2016–2023).

		BOTH GENDER	MALE	FEMALE
Study Variable	Categories	OR(95%CI)	OR(95%CI)	OR(95%CI)
Age groups (years)	40–49	Reference	Reference	Reference
	50–64	1.77 (1.55–2.02)	1.75 (1.52–2.02)	1.83 (1.29–2.6)
	65–79	2.51 (2.2–2.86)	2.47 (2.14–2.85)	2.77 (1.94–3.94)
	≥80	3.87 (3.38–4.42)	3.8 (3.29–4.39)	4.72 (3.21–6.94)
Cocaine use		0.7 (0.62–0.8)	0.71 (0.62–0.8)	0.66 (0.43–1.01)
Cannabinoids use		0.85 (0.75–0.97)	0.87 (0.76–1)	0.65 (0.41–1.04)
Opioid dependence		0.79 (0.7–0.89)	0.82 (0.72–0.94)	0.54 (0.36–0.82)
Tobacco use		0.84 (0.81–0.88)	0.85 (0.81–0.88)	0.84 (0.73–0.97)
Charlson Comorbidity Index		1.3 (1.28–1.32)	1.29 (1.28–1.31)	1.39 (1.32–1.46)
Pneumonia		1.41 (1.35–1.47)	1.41 (1.35–1.47)	1.46 (1.24–1.73)
Asthma		0.78 (0.7–0.87)	0.78 (0.69–0.88)	0.81 (0.63–1.03)
Sleep apnea		0.67 (0.63–0.7)	0.66 (0.63–0.7)	0.71 (0.56–0.91)
Obesity		0.66 (0.63–0.69)	0.65 (0.62–0.68)	0.76 (0.64–0.91)
Depression		0.81 (0.74–0.88)	0.82 (0.75–0.9)	0.74 (0.61–0.89)
Anxiety		0.81 (0.74–0.88)	0.83 (0.75–0.91)	0.74 (0.6–0.91)
Personality disorders		0.76 (0.64–0.89)	0.89 (0.74–1.06)	0.48 (0.33–0.7)
External causes		0.95 (0.89–1.02)	0.97 (0.91–1.04)	0.76 (0.6–0.95)
COVID 19		1.8 (1.68–1.93)	1.79 (1.67–1.93)	1.89 (1.43–2.5)
Long-term use of steroid		0.89 (0.82–0.97)	0.9 (0.82–0.98)	0.83 (0.61–1.12)
Supplemental oxygen		1.31 (1.25–1.37)	1.31 (1.25–1.37)	1.3 (1.11–1.53)
Invasive mechanical ventilation		5.17 (4.83–5.53)	5.12 (4.77–5.5)	5.34 (4.26–6.68)
Non–invasive mechanical ventilation		1.89 (1.78–2)	1.93 (1.81–2.05)	1.58 (1.3–1.91)
ICU		1.94 (1.84–2.05)	1.9 (1.8–2.01)	2.5 (2.07–3)
Year of admission	2016	Reference	Reference	Reference
	2017	1.04 (0.97–1.11)	1.03 (0.96–1.1)	1.15 (0.87–1.5)
	2018	1.03 (0.97–1.1)	1.03 (0.96–1.11)	1 (0.76–1.31)
	2019	1.02 (0.96–1.09)	1.02 (0.96–1.1)	0.94 (0.71–1.23)
	2020	1.23 (1.15–1.31)	1.22 (1.15–1.31)	1.23 (0.94–1.6)
	2021	1.14 (1.07–1.22)	1.14 (1.06–1.21)	1.19 (0.92–1.53)
	2022	1.01 (0.95–1.08)	1.01 (0.95–1.08)	1 (0.77–1.28)
	2023	1.01 (0.95–1.08)	1.02 (0.95–1.08)	0.92 (0.71–1.17)
Gender	Female	Reference	Reference	Reference
	Male	1.06 (1–1.12)	NA	NA

OR: Odds Ratio. CI: confidence interval. NA: Not Available.

## Data Availability

According to the contract signed with the Spanish Ministry of Health and Social Services, which provided access to the databases from the Spanish National Hospital Database, we cannot share the databases with any other investigator, and we have to destroy the databases once the investigation has concluded. Consequently, we cannot upload the databases to any public repository. However, any investigator can apply for access to the databases by filling out the questionnaire available at https://www.sanidad.gob.es/estadEstudios/estadisticas/estadisticas/estMinisterio/SolicitudCMBD.htm (accessed on 16 December 2024). All other relevant data are included in the paper.

## Supplementary Materials

The following supporting information can be downloaded at: https://www.mdpi.com/article/10.3390/jcm14176045/s1, Table S1: Diagnosis analyzed with their corresponding ICD10 codes; Table S2: Clinical characteristics and hospital outcomes in alcohol use disorders (AUD) in adults with COPD in Spain according to gender (2016–2023).

## Abbreviations

The following abbreviations are used in this manuscript:

COPD	Chronic Obstructive Pulmonary Disease
MBDA	Mental and Behavioral Disorders due to substance Abuse
AUD	Alcohol Use Disorder
SNHDD	Spanish National Hospital Discharge Database
HIV	Human Immunodeficiency Virus
ICU	Intensive Care Units
ICD-10-CM	International Classification of Diseases, 10th Revision, Clinical Modification
IHM	In-Hospital Mortality
OSA	Obstructive Sleeping Apnea
OAT	Opioid Agonist Treatment
