# Supplementary material for: Nationwide Trends and Outcomes of Alcohol Use Disorders in COPD Hospitalizations in Spain, 2016–2023
Source: J Clin Med. 2025 Aug 26;14(17):6045. doi: 10.3390/jcm14176045 (PMC12429768; doi:10.3390/jcm14176045)
Supplement: Supplementary file 1 [file jcm-14-06045-s001.zip › jcm-3776543-supplementary.pdf]

Table S1. Diagnosis analyzed with their corresponding ICD10 codes.

CONDITION	ICD 10 CODES
COPD	J40 A J44, Y J47
Mental and behavioural disorders due to use of alcohol	F10
Mental and behavioural disorders due to use of cocaine	F14
Mental and behavioural disorders due to use of cannabinoids	F12
Mental and behavioural disorders due to use of opioids	F11
Tobacco use	F17, Z72.0, Z87.891, T65.2
Obesity	E66.09; E66.1 E66.3; E66.8 E66.9 E66.2; E66.01
Depression	F32; F33
Anxiety	F40; F41
Specific personality disorders	F60
External causes of morbidity and mortality	S00 to T14.8x, T14.90, V00 to X58, X60-X84 AND T14.91
COVID 19	U071
Dependence on supplemental oxygen	Z99.81
Long-term use of steroid	Z79.5
Asthma	J45
Sleep apnea	G47.3 toG47.39
Non-invasive mechanical ventilation	5A09357, 5A09457, or 5A09557
Invasive mechanical ventilation	5A1935Z 5A1945Z, 5A1955Z,
Pneumonia	J13 to J18 and J95.851

Table S2. Clinical characteristics and hospital outcomes in alcohol use disorders (AUD) in in adults with COPD in Spain according to gender (2016-2023).

		MALE	FEMALE	P
		N (%)	N (%)	
Number of COPD hospital admissions		1929356	615795	<0.001
Number of AUD		248836(12.9)	24732(4.02)	<0.001
Age	Mean (SD)	68.93(10.39)	63.45(9.91)	<0,001
Age groups (years)	40-49	7867(25.61)	1803(10.44)	<0.001
	50-64	76749(25.4)	12067(8.91)	<0.001
	65-79	122988(14.14)	9343(4.03)	<0.001
	≥ 80	41232(5.68)	1519(0.66)	<0.001
Cocaine use		8639(3.47)	1464(5.92)	<0.001
Cannabinoids use		6539(2.63)	1086(4.39)	<0.001
Opioid dependence		6781(2.73)	1302(5.26)	<0.001
Tobacco use		209329(84.12)	20766(83.96)	0.514
Charlson Comorbidity Index		1.49(1.15)	1.07(0.98)	<0.001
Pneumonia		26187(10.52)	2312(9.35)	<0.001
Asthma		5526(2.22)	1775(7.18)	<0.001
Sleep apnea		34111(13.71)	1962(7.93)	<0.001
Obesity		36235(14.56)	3474(14.05)	0.028
Depression,		9121(3.67)	3013(12.18)	<0.001
Anxiety		8149(3.27)	2837(11.47)	<0.001
Personality disorders		2898(1.16)	1534(6.2)	<0.001
External causes		13042(5.24)	1915(7.74)	<0.001
Covid 19		7594(3.05)	651(2.63)	<0.001
Long-term use of steroid		8681(3.49)	1035(4.18)	<0.001
Supplemental oxygen		26982(10.84)	3051(12.34)	<0.001
Invasive mechanical ventilation		5927(2.38)	762(3.08)	<0.001
Non-invasive mechanical ventilation		10372(4.17)	1503(6.08)	<0.001
Admission to ICU		17904(7.2)	1933(7.82)	<0.001
IHM		18989(7.63)	1425(5.76)	<0.001

COPD: chronic obstructive pulmonary disease. AUD: alcohol use disorders. CCI: Charlson comorbidity index. External causes included ICD 10 codes for accidents, Injury and Intentional self-harm (See table S1). ICU: intensive care unit. IHM: in-hospital mortality. P value for difference by gender.
